# Comprehensive *In Silico* Characterization and Expression Profiling of *TCP* Gene Family in Rapeseed

**DOI:** 10.3389/fgene.2021.794297

**Published:** 2021-11-17

**Authors:** Yunfei Wen, Ali Raza, Wen Chu, Xiling Zou, Hongtao Cheng, Qiong Hu, Jia Liu, Wenliang Wei

**Affiliations:** ^1^ College of Agriculture, Yangtze University, Jingzhou, China; ^2^ Key Laboratory for Biological Sciences and Genetic Improvement of Oil Crops, Oil Crops Research Institute of Chinese Academy of Agricultural Sciences, Ministry of Agriculture and Rural Affairs, Wuhan, China; ^3^ Fujian Provincial Key Laboratory of Crop Molecular and Cell Biology, Center of Legume Crop Genetics and Systems Biology/College of Agriculture, Oil Crops Research Institute, Fujian Agriculture and Forestry University (FAFU), Fuzhou, China

**Keywords:** abiotic stress, *cis*-elements, evolution, genomics, miRNA, phytohomones, segmental duplication

## Abstract

TCP proteins are plant-specific transcription factors that have multipurpose roles in plant developmental procedures and stress responses. Therefore, a genome-wide analysis was performed to categorize the *TCP* genes in the rapeseed genome. In this study, a total of 80 *BnTCP* genes were identified in the rapeseed genome and grouped into two main classes (PCF and CYC/TB1) according to phylogenetic analysis. The universal evolutionary analysis uncovered that *BnTCP* genes had experienced segmental duplications and positive selection pressure. Gene structure and conserved motif examination presented that Class I and Class II have diverse intron-exon patterns and motifs numbers. Overall, nine conserved motifs were identified and varied from 2 to 7 in all *TCP* genes; and some of them were gene-specific. Mainly, Class II (PCF and CYC/TB1) possessed diverse structures compared to Class I. We identified four hormone- and four stress-related responsive *cis*-elements in the promoter regions. Moreover, 32 bna-miRNAs from 14 families were found to be targeting 21 *BnTCPs* genes. Gene ontology enrichment analysis presented that the *BnTCP* genes were primarily related to RNA/DNA binding, metabolic processes, transcriptional regulatory activities, etc. Transcriptome-based tissue-specific expression analysis showed that only a few genes (mainly *BnTCP9, BnTCP22, BnTCP25, BnTCP48, BnTCP52, BnTCP60, BnTCP66,* and *BnTCP74*) presented higher expression in root, stem, leaf, flower, seeds, and silique among all tested tissues. Likewise, qRT-PCR-based expression analysis exhibited that *BnTCP36, BnTCP39, BnTCP53, BnTCP59,* and *BnTCP60* showed higher expression at certain time points under various hormones and abiotic stress conditions but not by drought and MeJA. Our results opened the new groundwork for future understanding of the intricate mechanisms of *BnTCP* in various developmental processes and abiotic stress signaling pathways in rapeseed.

## Introduction

Transcription factors (TFs) contribute to plant growth and development, regulate gene expression, and play a significant role in several cellular/biological processes (Hoang et al., 2017; Karaaslan et al., 2020). The Teosinte Branched 1, Cycloidea, and Proliferating Cell Factors (*TCP*) gene family is a group of plant-specific TFs limited to higher plants (Cubas et al., 1999). These TFs have numerous roles in controlling plant growth and developmental procedures by directing cell proliferation (Cubas et al., 1999; Martín-Trillo and Cubas, 2010). Notably, these TFs are categorized by an extremely conserved 59-amino-acid basic helix–loop–helix (bHLH) motif at the N-terminus nominated as the TCP domain (Aggarwal et al., 2010). This domain is accountable for nuclear targeting, DNA binding and also contributes to protein-protein interactions (Kosugi and Ohashi, 2002). Due to the dissimilarity in the TCP domain, *TCP* gene family members are grouped into two main classes, i.e., Class I (PCF) and Class II (CYC/TB1 and CIN sub-classes) (Navaud et al., 2007; Viola et al., 2012). Along with the TCP domain, several members of Class II retain 18–20-residue arginine-rich motifs (Viola et al., 2012; Sarvepalli and Nath, 2018). This supposed R domain was anticipated to develop a hydrophilic-helix or α-coiled-coil structure that facilitates the protein-protein interactions in plants (Cubas et al., 1999).

Over the past few years, *TCP* gene families have been reported in several plant species. For example, 16 *TCP* members in Moso bamboo (*Phyllostachys edulis*) (Liu et al., 2018); 18 *TCP* members in sweet potato (*Ipomoea batatas* L.) (Ren et al., 2021); 23 *TCP* members in orchids (*Phalaenopsis equestris*) (Lin et al., 2016); 24 *TCP* members in *Arabidopsis thaliana* (Yao et al., 2007); 27 *TCP* members in cucumber (*Cucumis sativus* L.) (Wen et al., 2020); 28 *TCP* members in rice (*Oryza sativa* L.) (Yao et al., 2007); 38 *TCP* members in cotton (*Gossypium raimondii* L.) (Ma et al., 2014); 39 *TCP* members in turnips (*Brassica rapa* ssp. rapa) (Du et al., 2017); 63 *TCP* members in *Brassica juncea* var. tumida (He et al., 2020), 73 *TCP* members in allotetraploid cotton (*Gossypium hirsutum* L.) (Yin et al., 2018), etc. Several studies have shown that *TCP* genes play significant roles in plant growth and developmental processes, including trichome formation (Wang et al., 2013), seed germination (Zhang et al., 2019), petal development (van Es et al., 2018), floral development (Li et al., 2019), shoot branching (Braun et al., 2012), and leaf anatomical morphology (Ren et al., 2021). These findings indicate the diverse role of *TCP* genes in regulating plant developmental processes.

Likewise, several *TCP* genes expression is also regulated by various hormones and abiotic stress treatments. For instance, in cotton, the *GhTCP14* gene is considered a vital switch in an auxin-facilitated extension of fiber cells (Wang et al., 2013). In *A. thaliana*, the expression level of *AtTCP15* was induced by gibberellic acid (Gastaldi et al., 2020), and *AtTCP1* was positively regulated by brassinosteroids treatment (Gao et al., 2015). Furthermore, the different *TCP* genes also participate in ethylene, strigolactone, and cytokinins signaling pathways (Braun et al., 2012; van Es et al., 2018). Under abiotic stress conditions, the expression level of *AtTCP15* was induced by heat stress (Wu et al., 2016), and *TCP20/22* was positively regulated by the circadian clock in *A. thaliana* (Wu et al., 2016). In cotton, several *GhTCP* genes were significantly up-regulated under heat, salt, and drought stresses (Yin et al., 2018). In cucumber, many *CsTCP* genes significantly responded to temperature and photoperiod and were also induced by gibberellic acid and ethylene treatments (Wen et al., 2020). *TCP* genes are also expressed in different tissues, such as many *TCP* genes mainly expressed in flower, leaf, and stem, flower, bud differentiation, and gametophyte development in *Prunus mume* (Zhou et al., 2016).

Rapeseed (*Brassica napus* L.) is the second most crucial oilseed crop and possesses a complex genome. However, its growth and productivity are significantly affected at physiological, biochemical, and molecular levels in response to various plant hormones and abiotic stress conditions (Raza et al., 2021a; He et al., 2021; Raza, 2021; Su et al., 2021). To date, the *TCP* gene family and key *TCP* genes responding to abiotic stress have not been fully characterized in rapeseed. Consequently, this is the first genome-wide study to identify the *TCP* genes in the rapeseed genome. To boost our understanding into *TCP* genes evolution in rapeseed, their phylogenetic relationships, synteny study, gene structures, conserved motifs, *cis*-elements, miRNA predictions, and functional annotation were evaluated. Additionally, their expression profile in numerous tissues/organs and under various phytohormones and abiotic stress conditions were largely appraised, which deeply improved our understanding of the *TCP* genes in rapeseed.

## Material and Methods

### Identification and Characterization of *TCP* Genes in Rapeseed

As described in our recent studies (Raza et al., 2021a; Su et al., 2021), two methods were used to recognize *TCP* genes in the *B. napus* genome, i.e., BLASTP and the Hidden Markov Model (HMM). The genome of *B. napus* (ZhongShuang 11; ZS11 variety) was downloaded from the BnPIR database (http://cbi.hzau.edu.cn/bnapus/index.php). For BLASTP, the amino acid sequences of 24 *AtTCPs* were used as a query with an e-value set to 1e^−5^. The amino acid sequences of 24 *AtTCPs* were attained from the TAIR *Arabidopsis* genome database (http://www.arabidopsis.org/). Secondly, the local software HMMER 3.1 (http://www.hmmer.org/) was used to search the *TCP* genes with default constraints. Then, the HMM file of the TCP domain (PF03634) was downloaded from the Pfam protein domain database (http://pfam.xfam.org/). Finally, 80 *BnTCP* genes were found by merging the two processes in the rapeseed genome. Likewise, *TCP* genes were also identified in *Brassica rapa* and *Brassica oleracea* genomes using the same method. Their genome sequences were downloaded from JGI Phytozome 12.0 database (https://phytozome.jgi.doe.gov/pz/portal.html).

The physico-chemical properties of *BnTCPs* were evaluated with the help of ProtParam (http://web.expasy.org/protparam/). The subcellular localization of BnTCP proteins was prophesied from the WoLF PSORT server (https://wolfpsort.hgc.jp/). Genes structures were created *via* TBtools software (V 1.068; https://github.com/CJ-Chen/TBtools). The conserved motifs of BnTCP protein sequences were recognized employing the MEME website (https://meme-suite.org/meme/db/motifs).

### Phylogenetic Tree and Synteny Analysis of BnTCP Proteins

To explore the evolutionary relationship of the *BnTCP*s, a phylogenetic tree between *B. napus, B. oleracea, B. rapa,* and *A. thaliana* protein sequences were constructed. The sequence alignment was executed using MEGA 7 software (https://megasoftware.net/home). The neighbor-joining process was accomplished to create a phylogenetic tree with 1,000 bootstrap replicates using the Evolview v3 website (https://www.evolgenius.info/evolview) to display the phylogenetic tree. Synteny relationships of *TCP* genes between *B. napus, B. oleracea, B. rapa,* and *A. thaliana* were performed using the python-package, JCVI (https://github.com/tanghaibao/jcvi) and Multiple Collinearity Scan Toolkit (MCScanX; https://github.com/wyp1125/MCScanX). The Ka/Ks ratios of all *TCPs* were calculated using KaKs_Calculator 2.0 (https://sourceforge.net/projects/kakscalculator2/).

### Analysis of *Cis*-Regulatory Elements in the *BnTCPs* Promoters

To investigate the putative *cis*-elements in the *BnTCPs* promoters, we extracted the 2Kb sequence upstream of start codons in the *B. napus* genome. Moreover, the promoter sequence of each gene was examined applying the PlantCARE website (http://bioinformatics.psb.ugent.be/webtools/plantcare/html/), and the figure was drawn with the help of TBtools software.

### Prediction of Putative miRNA Targeting *BnTCP* Genes and GO Annotation Analysis

The CDS of BnTCPs was employed to distinguish possible target miRNAs in the psRNATarget database (https://www.zhaolab.org/psRNATarget/home) with default parameters. The interaction network figure between the miRNAs and BnTCP genes was constructed with Cytoscape software (V3.8.2; https://cytoscape.org/download.html). Gene ontology (GO) annotation analysis was accomplished by submitting all BnTCPs protein sequences to the eggNOG website (http://eggnog-mapper.embl.de/). At the same time, GO enrichment analysis was performed with TBtools software.

### Expression Profiling of *BnTCP* Genes in Diverse Tissues

For tissue-specific expression profiling, the rapeseed RNA-seq data was downloaded from the National Genomics Data Center (https://ngdc.cncb.ac.cn/gsa/; BioProject ID: PRJCA001495). The data were analyzed as described in a recent study (Raza et al., 2021a). In short, Cuffquant and Cuffnorm were used to produce normalized counts in transcripts per million (TPM) values. Due to the large difference in the expression profiles, we used the log10 method to calculate the expression results to better visualize differently expressed genes in diverse tissues. Based on log10 values, the expression heat map was created using GraphPad Prism 9.0.0 software (https://www.graphpad.com/).

### Plant Material and Stress Conditions

A widely used variety, ZhongShuang 11 (ZS11), was used for stress treatments. Seeds of ZS11 were provided by the OCRI-CAAS, Wuhan, China. The stress treatments were carried out as described in our recent work (Raza et al., 2021a). All the treatments were executed with three biological replicates. All the samples were immediately frozen in liquid nitrogen and were stored at −80°C for further experimentation.

### RNA Extraction and qRT-PCR Analysis

Total RNA was extracted using TransZol Up Plus RNA Kit (TransGen Biotech, Beijing, China), and cDNA was synthesized using cDNA SuperMix kit (TransGen Biotech, Beijing, China) according to developer instructions. The complete data of qRT-PCR reaction has been explained in our recent work (Raza et al., 2021a). The expression data were investigated using the 2^−ΔΔCT^ method as described by Wang et al. (2014). All the primers used for qRT-PCR are listed in Supplementary Table S1. The graphs were developed using GraphPad Prism 9.0.0 software.

## Results

### Identification and Characterization of *TCP* Genes in Rapeseed

To identify *TCP* family genes in rapeseed, 24 AtTCP protein sequences were used as queries for a BlastP search against the rapeseed genome. As a result, a sum of 80 *BnTCP* genes was identified with the TCP domain (Table 1). Hereafter, these genes are named as “*BnTCP1*–*BnTCP80.*” Among which 38 genes were positioned in the A subgenome, and 42 genes were positioned in the C subgenome (Table 1). Comprehensive data of the 80 *BnTCP* genes are shown in Table 1. Briefly, gene length varied from 531 bp (*BnTCP10*) to 9,673 bp (*BnTCP40*), the CDS length varied from 531 bp (*BnTCP10*) to 5,193 bp (*BnTCP40*), and the amino acid length varied from 176 (*BnTCP10*) to 1,730 (*BnTCP40*) amino acids. The number of exons varied from one (*BnTCP3*–*BnTCP6, BnTCP8*–*BnTCP10, BnTCP12*–*BnTCP15*) to 11 (*BnTCP40*) (Table 1). Notably, only two genes (*BnTCP40* and *BnTCP66*) had the highest number of introns (11 and 8, respectively), and several genes lack introns, such as *BnTCP3-BnTCP6, BnTCP8*–*BnTCP10, BnTCP12*–*BnTCP15* (Table 1). The forecasted molecular weights of the 80 BnTCP proteins extended from 18.60 kDa (BnTCP10) to 194.78 kDa (BnTCP40), and the isoelectric points prolonged from 5.31 (BnTCP28/45) to 11.12 (BnTCP49). The differences in molecular weights and isoelectric points are mainly due to the unusual high content of basic amino acids and post-translational modifications. The results of *in silico* subcellular localization prophesied that two BnTCP proteins were located on the peroxisome, four BnTCP proteins on the chloroplast, seven BnTCP proteins on the mitochondria, and remaining 67 BnTCP proteins were located on the nucleus (Table 1). Meanwhile, 24 genes (*AtTCP1*–*AtTCP24*) from *A. thaliana*, 39 genes (*BraTCP1*–*BraTCP39*) from *B. rapa*, and 40 genes (*BoTCP1*–*BoTCP41*) from *B. oleracea* genomes were also identified (Supplementary Table S2).

**TABLE 1:** Information of the identified *TCP* genes in rapeseed genome.

Gene ID	Gene name	Genomic position (bp)	Gene length (bp)	CDS length (bp)	Exon	Intron	Protein length (aa)	Molecular weight (kDa)	Theoretical isoelectric point (pi)	Subcellular localization
BnaA01T0093200ZS	*BnTCP1*	A01: 5350331–5352930: −	2,600	1,077	2	1	358	39.2194	8.32	Nucleus
BnaA01T0326500ZS	*BnTCP2*	A01: 30234583–30236602: −	2,020	1,284	4	3	427	48.7818	7.19	Nucleus
BnaA01T0351100ZS	*BnTCP3*	A01: 31838550–31839623: −	1,074	1,074	1	0	357	38.9368	7.45	Nucleus
BnaA02T0029000ZS	*BnTCP4*	A02: 1746997–1747686: +	690	690	1	0	229	23.9396	10.2	Nucleus
BnaA02T0147800ZS	*BnTCP5*	A02: 8342653–8343498: +	846	846	1	0	281	30.2123	5.42	Nucleus
BnaA02T0165200ZS	*BnTCP6*	A02: 9824501–9825541: −	1,041	1,041	1	0	346	39.2804	5.48	Nucleus
BnaA02T0176600ZS	*BnTCP7*	A02: 10751996–10753110: −	1,115	1,011	2	1	336	38.2344	9.04	Nucleus
BnaA02T0183300ZS	*BnTCP8*	A02: 11226562–11227527: +	966	966	1	0	321	33.8707	7.73	Nucleus
BnaA02T0196800ZS	*BnTCP9*	A02: 12284709–12285833: +	1,125	1,125	1	0	374	39.0581	8.99	Nucleus
BnaA02T0389800ZS	*BnTCP10*	A02: 33966392–33966922: −	531	531	1	0	176	18.6008	8.29	Mitochondria
BnaA02T0391800ZS	*BnTCP11*	A02: 34141978–34143043: −	1,066	906	2	1	301	33.862	7.76	Nucleus
BnaA03T0030500ZS	*BnTCP12*	A03: 1438953–1439693: +	741	741	1	0	246	27.3701	7.02	Nucleus
BnaA03T0032200ZS	*BnTCP13*	A03: 1501650–1502354: +	705	705	1	0	234	24.2451	10.87	Nucleus
BnaA03T0221500ZS	*BnTCP14*	A03: 11512211–11513155: −	945	945	1	0	314	33.4158	10.47	Nucleus
BnaA03T0286400ZS	*BnTCP15*	A03: 15203307–15204224: +	918	918	1	0	305	34.0894	7.89	Nucleus
BnaA03T0339700ZS	*BnTCP16*	A03: 18046255–18048379: +	2,125	1,218	3	2	405	44.1964	8.02	Nucleus
BnaA03T0354000ZS	*BnTCP17*	A03: 18816141–18817922: +	1,782	1,278	4	3	425	48.4559	8.5	Nucleus
BnaA03T0405400ZS	*BnTCP18*	A03: 21941344–21942444: −	1,101	1,101	1	0	366	40.739	6.68	Nucleus
BnaA03T0447400ZS	*BnTCP19*	A03: 24280135–24281286: −	1,152	1,152	1	0	383	42.3337	7.69	Nucleus
BnaA04T0122600ZS	*BnTCP20*	A04: 13913091–13913765: +	675	675	1	0	224	24.7676	7.82	Mitochondria
BnaA05T0054400ZS	*BnTCP21*	A05: 2969609–2971094: −	1,486	975	1	0	324	34.5098	10.18	Nucleus
BnaA05T0129300ZS	*BnTCP22*	A05: 7820651–7821986: +	1,336	1,047	2	1	348	38.6262	6.85	Peroxisome
BnaA05T0402900ZS	*BnTCP23*	A05: 39177839–39180026: −	2,188	1,227	2	1	408	44.3164	7.55	Nucleus
BnaA06T0174300ZS	*BnTCP24*	A06: 11013702–11015537: −	1,836	1,437	1	0	478	51.5508	7.21	Nucleus
BnaA06T0320300ZS	*BnTCP25*	A06: 40732993–40733748: +	756	756	1	0	251	27.1051	10.33	Nucleus
BnaA06T0386200ZS	*BnTCP26*	A06: 44783008–44783931: +	924	924	1	0	307	32.3907	7.96	Nucleus
BnaA07T0270200ZS	*BnTCP27*	A07: 25405486–25406442: −	957	957	1	0	318	33.7336	8.08	Mitochondria
BnaA07T0280800ZS	*BnTCP28*	A07: 26138757–26139815: +	1,059	1,059	1	0	352	39.9883	5.31	Nucleus
BnaA07T0291800ZS	*BnTCP29*	A07: 26854045–26855100: −	1,056	1,056	1	0	351	39.9273	7.2	Nucleus
BnaA07T0312100ZS	*BnTCP30*	A07: 27924720–27925685: +	966	966	1	0	321	34.0659	7.47	Nucleus
BnaA07T0330100ZS	*BnTCP31*	A07: 29148894–29149970: +	1,077	1,077	1	0	358	37.3864	7.47	Nucleus
BnaA08T0015500ZS	*BnTCP32*	A08: 1202449–1203435: +	987	987	1	0	328	35.6779	7.24	Nucleus
BnaA08T0205600ZS	*BnTCP33*	A08: 22895827–22896753: +	927	834	2	1	277	30.8253	6.95	Nucleus
BnaA09T0037700ZS	*BnTCP34*	A09: 2358891–2359727: −	837	837	1	0	278	29.6245	6.63	Nucleus
BnaA09T0073000ZS	*BnTCP35*	A09: 4327191–4327838: −	648	648	1	0	215	23.0186	9.87	Chloroplast
BnaA09T0175800ZS	*BnTCP36*	A09: 11575172–11576356: +	1,185	1,185	1	0	394	41.4311	6.46	Nucleus
BnaA09T0412200ZS	*BnTCP37*	A09: 47278416–47282262: +	3,847	957	3	2	318	35.2881	8.13	Nucleus
BnaA10T0253900ZS	*BnTCP38*	A10: 24336051–24336761: −	711	711	1	0	236	24.7343	8.55	Nucleus
BnaC01T0114300ZS	*BnTCP39*	C01: 7792345–7794974: −	2,630	1,080	2	1	359	39.2785	8.32	Nucleus
BnaC01T0126300ZS	*BnTCP40*	C01: 8666079–8675751: +	9,673	5,193	11	10	1730	194.7861	8.16	Nucleus
BnaC01T0404000ZS	*BnTCP41*	C01: 46796515–46798601: −	2,087	1,293	4	3	430	49.2565	7.4	Nucleus
BnaC01T0435000ZS	*BnTCP42*	C01: 50033768–50035625: −	1,858	1,089	2	1	362	39.202	7.25	Nucleus
BnaC02T0032000ZS	*BnTCP43*	C02: 2159588–2160265: +	678	678	1	0	225	23.5673	9.85	Nucleus
BnaC02T0189600ZS	*BnTCP44*	C02: 15598917–15599783: +	867	867	1	0	288	30.9993	6.15	Nucleus
BnaC02T0211700ZS	*BnTCP45*	C02: 18337482–18338522: −	1,041	1,041	1	0	346	39.4164	5.31	Nucleus
BnaC02T0229100ZS	*BnTCP46*	C02: 20787472–20790829: −	3,358	1,005	4	3	334	38.0556	7.95	Nucleus
BnaC02T0241600ZS	*BnTCP47*	C02: 22073752–22074708: +	957	957	1	0	318	33.7857	7.71	Nucleus
BnaC02T0261700ZS	*BnTCP48*	C02: 24613854–24614969: +	1,116	1,116	1	0	371	38.7778	9.38	Nucleus
BnaC02T0521300ZS	*BnTCP49*	C02: 62454902–62455594: −	693	693	1	0	230	24.9241	11.12	Nucleus
BnaC02T0524000ZS	*BnTCP50*	C02: 62771638–62772713: −	1,076	906	2	1	301	33.7318	6.72	Nucleus
BnaC03T0037100ZS	*BnTCP51*	C03: 1909961–1910695: +	735	735	1	0	244	27.341	6.91	Nucleus
BnaC03T0039100ZS	*BnTCP52*	C03: 2018328–2019035: +	708	708	1	0	235	24.258	10.87	Nucleus
BnaC03T0260500ZS	*BnTCP53*	C03: 16121718–16122599: −	882	882	1	0	293	30.929	10.32	Nucleus
BnaC03T0344300ZS	*BnTCP54*	C03: 23421941–23422903: +	963	963	1	0	320	35.7522	7.08	Nucleus
BnaC03T0408900ZS	*BnTCP55*	C03: 28003605–28005738: +	2,134	1,215	3	2	404	44.0973	8.04	Nucleus
BnaC03T0429200ZS	*BnTCP56*	C03: 29713443–29715268: +	1,826	1,278	4	3	425	48.6133	8.66	Nucleus
BnaC03T0620400ZS	*BnTCP57*	C03: 59527128–59528955: +	1,828	1,422	1	0	473	51.1025	7.44	Nucleus
BnaC03T0663600ZS	*BnTCP58*	C03: 64144410–64145333: −	924	924	1	0	307	34.4323	8.09	Nucleus
BnaC04T0060500ZS	*BnTCP59*	C04: 5408796–5409773: −	978	978	1	0	325	34.6679	10.03	Chloroplast
BnaC04T0168800ZS	*BnTCP60*	C04: 15546097–15547417: +	1,321	1,047	2	1	348	38.6283	7.04	Peroxisome
BnaC04T0410600ZS	*BnTCP61*	C04: 53526218–53526877: +	660	660	1	0	219	23.7855	6.89	Mitochondria
BnaC05T0259700ZS	*BnTCP62*	C05: 21147596–21148549: −	954	954	1	0	317	35.0458	8.13	Nucleus
BnaC05T0413400ZS	*BnTCP63*	C05: 46522363–46523833: −	1,471	1,248	3	2	415	47.8023	8.78	Nucleus
BnaC05T0452400ZS	*BnTCP64*	C05: 50135134–50137315: −	2,182	1,215	2	1	404	44.2776	8.38	Nucleus
BnaC05T0566500ZS	*BnTCP65*	C05: 57728974–57732707: +	3,734	1,047	3	2	348	38.7422	7.42	Nucleus
BnaC05T0569300ZS	*BnTCP66*	C05: 57878862–57886495: +	7,634	1,944	9	8	647	72.5415	8.6	Chloroplast
BnaC06T0305200ZS	*BnTCP67*	C06: 40942818–40943765: −	948	948	1	0	315	33.6966	8.08	Mitochondria
BnaC06T0321700ZS	*BnTCP68*	C06: 42719251–42720315: +	1,065	1,065	1	0	354	40.2017	5.86	Nucleus
BnaC06T0335100ZS	*BnTCP69*	C06: 44122410–44123480: −	1,071	1,071	1	0	356	40.574	5.56	Nucleus
BnaC06T0353200ZS	*BnTCP70*	C06: 45538660–45539631: −	972	972	1	0	323	36.4448	8.94	Nucleus
BnaC06T0362700ZS	*BnTCP71*	C06: 46363762–46364718: +	957	957	1	0	318	34.0369	7.65	Nucleus
BnaC06T0386600ZS	*BnTCP72*	C06: 48098081–48099548: +	1,468	1,068	1	0	355	37.2392	7.17	Nucleus
BnaC07T0302100ZS	*BnTCP73*	C07: 44542787–44543746: −	960	960	1	0	319	33.6842	8	Nucleus
BnaC07T0373600ZS	*BnTCP74*	C07: 49902745–49903473: −	729	729	1	0	242	26.0729	10.11	Nucleus
BnaC07T0378100ZS	*BnTCP75*	C07: 50224221–50225315: −	1,095	1,095	1	0	364	40.5408	6.68	Nucleus
BnaC07T0422500ZS	*BnTCP76*	C07: 53049841–53052375: −	2,535	1,125	2	1	374	41.2696	7.62	Nucleus
BnaC09T0023100ZS	*BnTCP77*	C09: 1586085–1587093: −	1,009	831	2	1	276	29.3745	6.75	Mitochondria
BnaC09T0063000ZS	*BnTCP78*	C09: 4101151–4101798: −	648	648	1	0	215	22.9824	9.87	Chloroplast
BnaC09T0197900ZS	*BnTCP79*	C09: 17390038–17391216: +	1,179	1,179	1	0	392	41.1277	6.51	Mitochondria
BnaC09T0567700ZS	*BnTCP80*	C09: 64952104–64952814: −	711	711	1	0	236	24.7502	7.81	Nucleus

In the genomic position, the positive (+) and negative (−) sign shows the presence of a gene on the positive and negative strand of that specific marker correspondingly.

### Phylogenetic Relationship of *TCP* Genes

**FIGURE 1:**
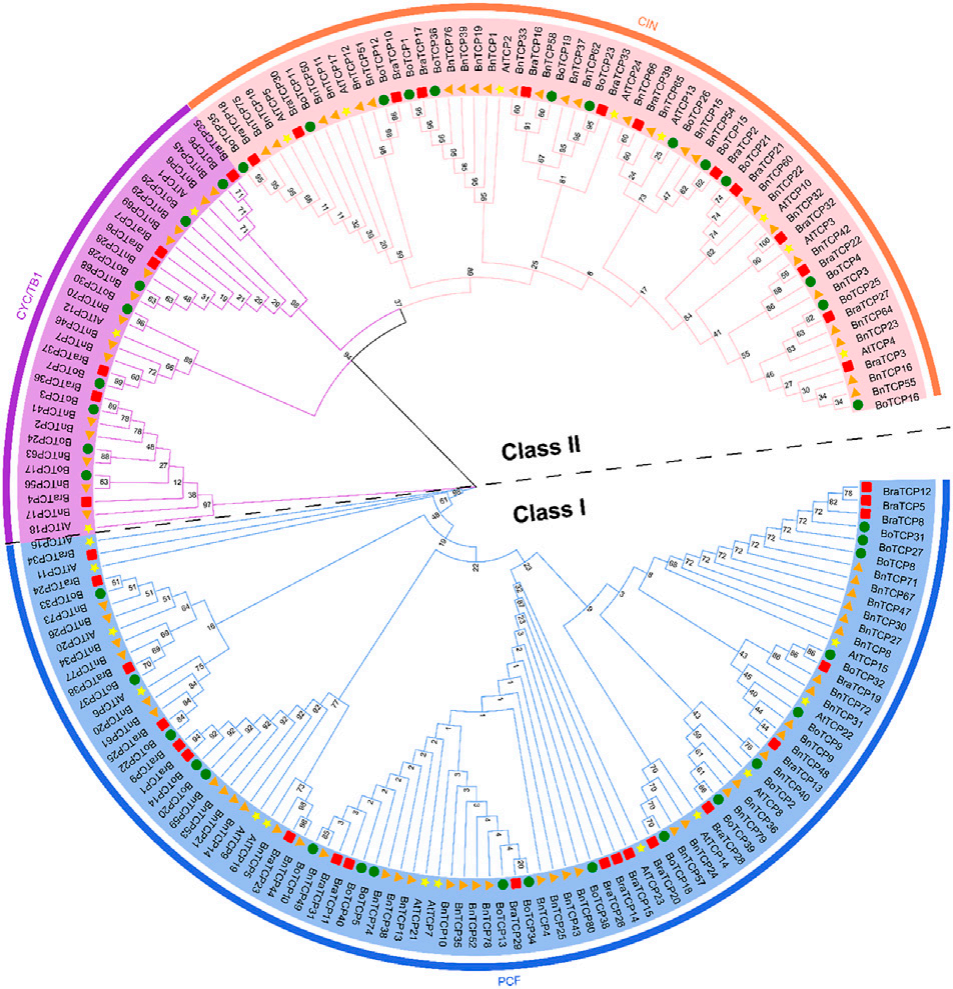
A neighbor-joining phylogenetic tree assessment of *TCP* genes from *B. napus, B. oleracea, B. rapa*, and *A. thaliana*. Overall, 80 *BnTCPs* from *B. napus* (orange triangle), 40 *BoTCPs* from *B. oleracea* (green circles), 39 *BraTCPs* from *B. rapa* (red boxes), and 24 *AtTCPs* from *A. thaliana* (yellow stars) were clustered into two major classes, denoted by exclusive colors.

To unreveal the evolutionary relationship between the *BnTCPs, BoTCPs, BraTCPs,* and *AtTCPs* genes, an unrooted phylogenetic tree was built by a multiple sequence alignment of the prophesied TCP protein sequences from *B. napus, B. oleracea, B. rapa,* and *A. thaliana* (Figure 1). Based on the standard classification of *Arabidopsis TCP* genes (Yao et al., 2007), the 184 *TCPs* genes from four plant species were distributed into two main classes, i.e., Class I (PCF) and Class II. Further, Class II was further divided into two sub-classes (CYC/TB1 and CIN) (Figure 1). The *TCPs* from the four plant species were scattered in nearly all clades, suggesting that the *TCP* family genes differentiated before the separation of these ancestral plant species. The findings discovered that Class I (PCF) was encompassed 91 *TCP* members (39 *BnTCPs*, 19 *BoTCP*s, 20 *BraTCPs,* and 13 *AtTCPs*). Class II contained a sum of 92 *TCP* members. Particularly, sub-class CYC/TB1 was comprised of 31 *TCP* members (14 *BnTCPs*, eight *BoTCP*s, six *BraTCPs,* and three *AtTCPs*), and CIN was comprised of 61 *TCP* members (27 *BnTCPs*, 13 *BoTCP*s, 13 *BraTCPs,* and eight *AtTCPs*) (Figure 1; Supplementary Table S3). In a nutshell, *TCPs* grouping into the same sub-class may retain corresponding functions. It is worth highlighting that *BnTCP* genes were almost equally scattered in both classes with homologs from other plant species (Figure 1; Supplementary Table S3). Mainly, Class I (PCF) was observed to have a greater number of *BnTCPs* (39), followed by CIN (27) and CYC/TB1 (14). Moreover, it was observed that the *BnTCPs* have a more closely phylogenetic relationship with the *BoTCPs* and *BraTCPs* in each class.

### Chromosomal Locations and Synteny Evaluation of *TCP* Genes

**FIGURE 2:**
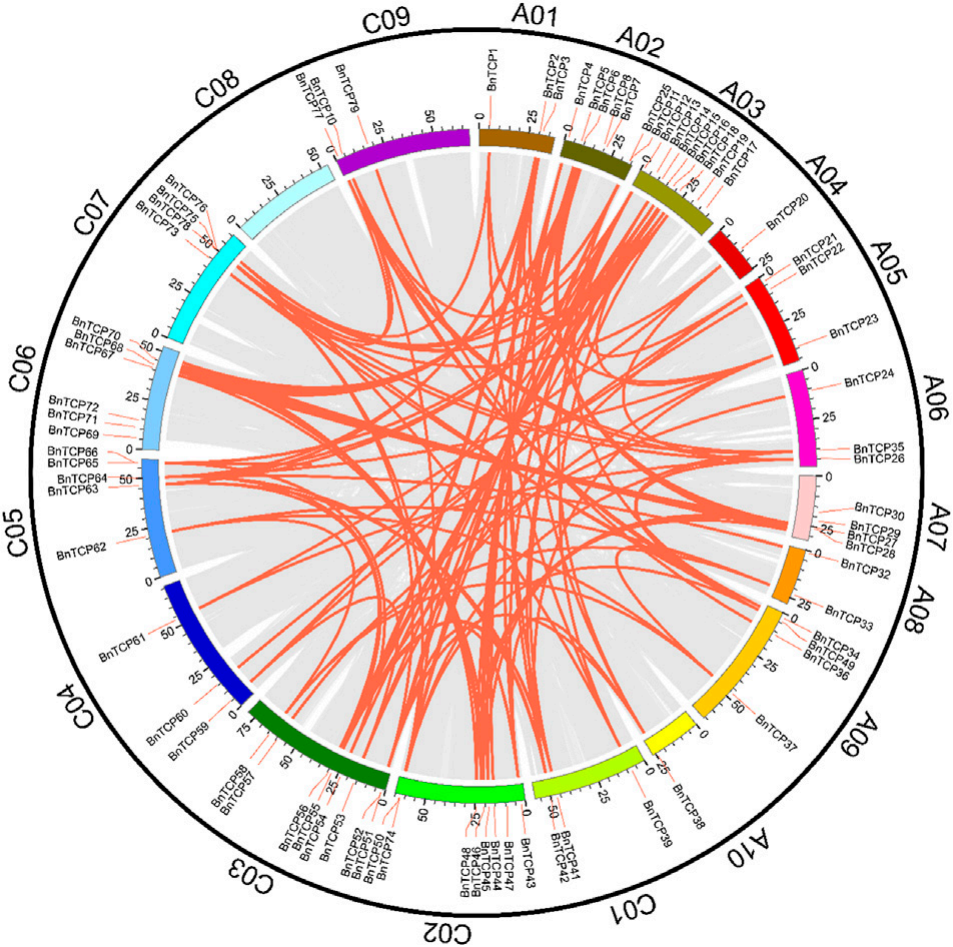
Chromosomal locations and inter-chromosomal relations of *BnTCP* genes. Grey lines in the background present all syntenic blocks in the *B. napus* genome, and the red lines present syntenic *BnTCPs* gene pairs.

The chromosol location of 38 *BnTCPs* gene pairs was observed (Figure 2; Supplementary Table S4), and 18 out of 19 chromosomes held *BnTCPs* genes (Figure 2). Mainly, chromosomes A04 and A10 possessed only one gene, chromosome A08 possessed two genes, chromosomes A01, A05, A06, C01, C04, and C09 possessed three genes, chromosomes A07, A09, and C07 possessed four genes, chromosomes A02 and C05 possessed five genes, chromosome C06 possessed six genes, chromosomes C02 and C03 possessed eight genes. Chromosome A03 had the highest number of genes (10). Astonishingly, the C08 chromosome did not contain any *BnTCP* genes (Figure 2). The dataset displays that segmental duplications have contributed to the development of *BnTCP* genes (Supplementary Table S4). Notably, one putative paralog pair, i.e., *BnTCP16/BnTCP55*, was produced by tandem duplication (Supplementary Table S4). These results showed the large-scale segmental duplication events involved in the evolution of the *BnTCP* gene family in rapeseed.

**FIGURE 3:**
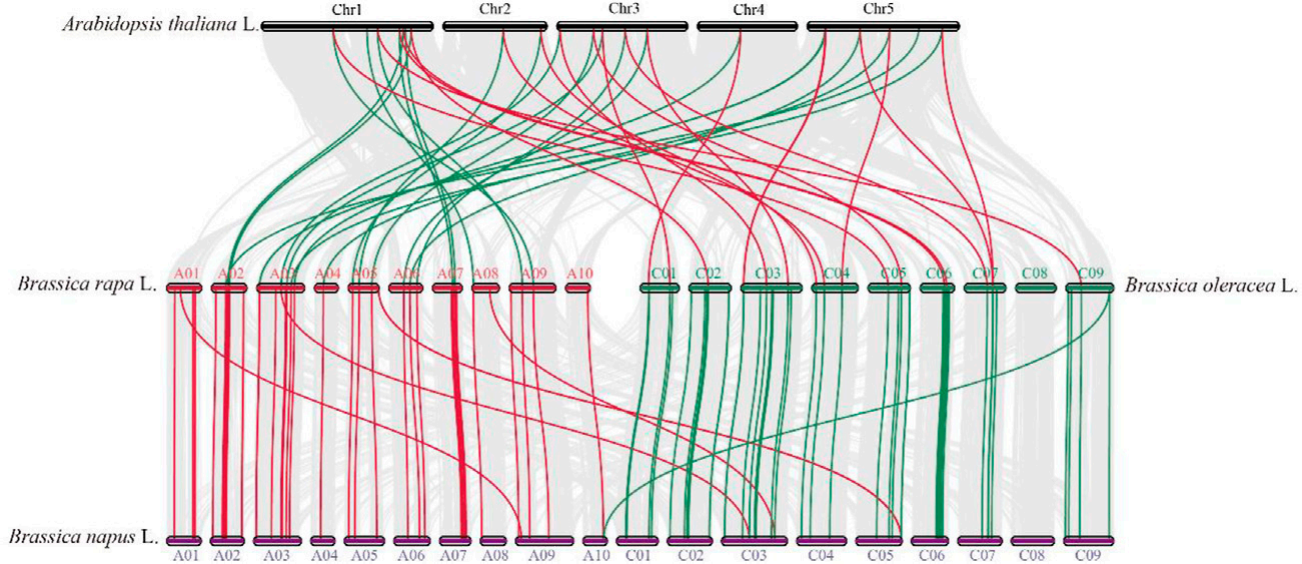
Collinearity analysis of *TCP* genes in *B. napus, B. rapa, B. oleracea,* and *A. thaliana* chromosomes. Grey lines in the background display the collinear blocks within *B. napus* and other plant genomes. However, the red and green lines signify the syntenic *TCP* gene pairs. Genes located on the *B. napus* A subgenome are syntenic with *B. rapa, B. oleracea,* and *A. thaliana*, while genes located on *B. napus* C subgenome are mainly syntenic with *B. rapa, B. oleracea,* and *A. thaliana.*

Collinearity analysis was used to appraise the evolutionary relationship of the *TCP* genes between *B. napus* and the other heritable plant species (Figure 3; Supplementary Table S4). In general, in the A subgenome, several *B. napus* genes showed syntenic networks with various *BraTCPs,* and *AtTCP*. Notably, one *BnTCP* gene at chromosome A10 also displayed a syntenic relationship with a *BoTCP* gene at chromosome C09. Likewise, in the C subgenome, many *B. napus* genes revealed syntenic connections with different *BraTCPs*, and *AtTCPs* (Figure 3). Mainly, three *BnTCP* genes at chromosome C03 and C05 also showed a syntenic relationship with three *BrTCP* genes at chromosome A03, A05 and A08 (Figure 3). On the other hand, several homologues of *A. thaliana, B. rapa,* and *B. oleracea* showed a syntenic alliance with *BnTCPs* genes in the rapeseed genome, suggesting that whole-genome or segmental duplication events represented a crucial role in the evolution of *BnTCPs* gene family in the rapeseed genome (Figure 3; Supplementary Table S4).

The Ka/Ks ratio is regarded as a considerable indicator in assessing the sequence evolution in terms of selection pressures and duplication types (Hurst, 2002). Hence, to comprehend the evolutionary history of the *BnTCPs*, the Ka, Ks, and Ka/Ks ratio was determined for *B. napus* and the other three plant species (Supplementary Table S4). The calculations disclosed that all of the duplicated *BnTCP* gene pairs had a Ka/Ks ratio of <1 (Supplementary Table S4), indicating that the *BnTCP* genes may have undergone intense purifying selective pressure during the evolution process (Supplementary Table S4). Similar outcomes were also examined in *B. rapa, B. oleracea,* and *A. thaliana* (Supplementary Table S4).

### Assessment of Gene Structures and Conserved Motifs of *BnTCP* Genes

**FIGURE 4:**
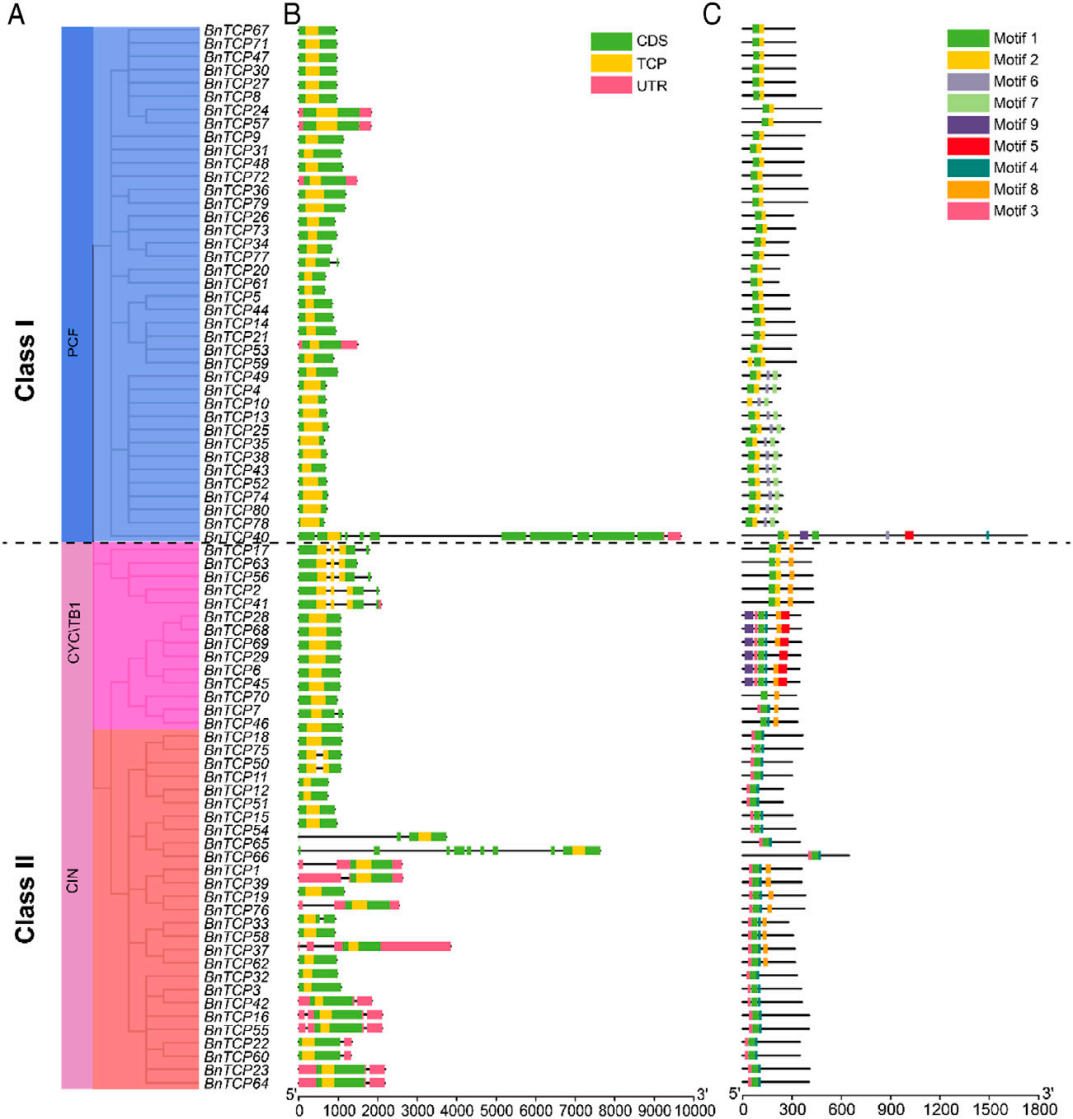
The gene structure and motif analysis of *BnTCPs* genes. **(A)** Based on phylogenetic relationships, the *BnTCPs* were clustered into two major classes. **(B)** Gene structure of *BnTCPs* genes. The pink color signifies UTR regions, the light green color denotes exon or CDS, the yellow color represents TCP domain, and the black horizontal line symbolizes introns. **(C)** Conserved motif compositions detected in *BnTCPs*. Different color boxes represent different motifs.

The exon-intron configurations of the *BnTCP* genes were examined to boost our knowledge of the evolution of the rapeseed *TCP* family genes. The results showed that the number of introns and exons ranged from 0 to 10 and 1–11, respectively (Figure 4B; Table 1). Overall, 55 genes have one exon and zero intron; 13 genes have two exons and one intron; five genes have three exons and two introns; five genes have four exons and three introns; one gene has nine exons and eight introns; and one gene has 11 exons and 10 introns (Figure 4; Table 1). Notably, Class I (PCF) and Class II (CIN and CYC/TB1) possessed almost similar structures except for a few genes (Figure 4B). Losses or gains of exons were discovered throughout the evolution of the *TCP* family genes. Our outcomes advised that *TCP* genes retained a comparatively constant exon-intron arrangement during the evolution of the rapeseed genome. Moreover, *BnTCP* gene members inside a Class had extremely matching gene structures, constant with their phylogenetic groups.

Furthermore, the full-length protein sequences were examined to identify their conserved motifs (Figure 4C). The conserved motif of the *TCP* genes varied from 2 to 7. Overall, nine conserved motifs were identified, and the complete data (such as name, sequence, width, and E-value) of discovered motifs are presented in Supplementary Table S5. The motif dispersals were also comparable within the Classes (Figure 4C). For example, motifs 1, 2, 6, and 7 were specific to Class I; however, motifs 6 were also present in some genes belonging to Class II (mainly CYC/TB1). In Class I, only one gene (*BnTCP40*) was found to have a greater number of motifs (seven). In Class II (mainly CYC/TB1), motifs 5 and 9 were specific and absent in other genes. Likewise, motifs 4, 8, and 3 were specific to Class II (CIN and CYC/TB1) (Figure 4C). In short, the class organization’s consistency was convincingly sustained by evaluating the conserved motifs composition, gene structures, and phylogenetic interactions, indicating that the TCP proteins have tremendously well-maintained amino acid remains and members within a class may possess parallel roles.

### Examination of *Cis*-Elements in Promoters of *BnTCP* Genes

**FIGURE 5:**
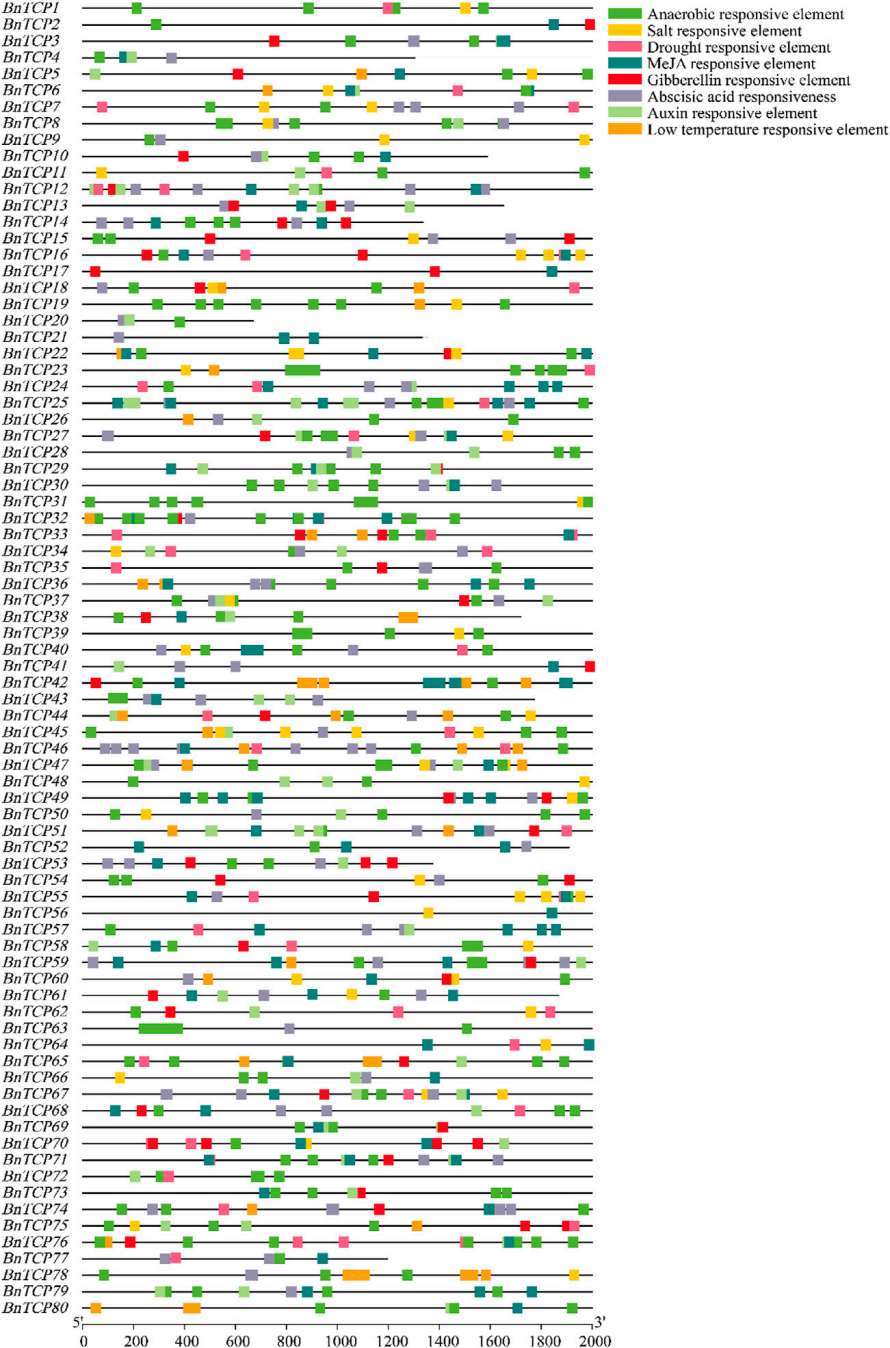
*Cis*-elements in the promoter regions of the *BnTCP* genes are linked with different hormone- and stress-responsive elements. Different color boxes show different identified elements. See Supplementary Table S6 for more information.

The *cis*-acting elements in the gene promoter generally regulate gene expression levels and functions. Therefore, *cis*-acting elements in *BnTCPs* promoter localities were analyzed to characterize the gene functions and regulatory roles. The comprehensive data of *cis*-elements are shown in Supplementary Table S6. Overall, we focused on and identified eight types of elements. Particularly, four phytohormone-correlated [(abscisic acid (ABA), auxin, methyl jasmonate (MeJA), and gibberellin (GA)] responsive elements consist of ABRE, TGA-element, TGACG-motif, CGTCA-motif, P-box, GARE-motif, TATC-box, were distinguished (Figure 5; Supplementary Table S6). Specifically, several phytohormone-correlated elements were anticipated to be limited to some genes and extensively dispersed (Figure 5; Supplementary Table S6), indicating the essential role of these genes in phytohormone-arbitration.

Additionally, we identified four stress-associated (salt, drought, low-temperature, and anaerobic) responsive elements, including TCA-element, MBS, LTR, ARE, suggesting their contribution to stress stimulation (Figure 5; Supplementary Table S6). TCA-element associated with salt stress responsiveness was mainly present in 38 *BnTCP* genes and widely distributed in four genes (*BnTCP16, BnTCP22, BnTCP45,* and *BnTCP55*) (Figure 5; Supplementary Table S6). Drought stress responsive element (MBS) was mainly present in 36 genes and widely circulated in 12 genes (Figure 5; Supplementary Table S6). Low-temperature responsive element (LTR) was particularly present in 26 genes and widely scattered in six genes (*BnTCP42, BnTCP44, BnTCP46, BnTCP65, BnTCP78,* and *BnTCP80*) (Figure 5; Supplementary Table S6). ARE element associated with anaerobic responsiveness was mainly present in 74 genes and largely dispersed in 35 genes (Figure 5; Supplementary Table S6). Generally, these findings recommended that *BnTCPs* expression profiles may differ under phytohormone and abiotic stress conditions.

### Genome-Wide Prediction of miRNA Targeting *BnTCP* Genes

**FIGURE 6:**
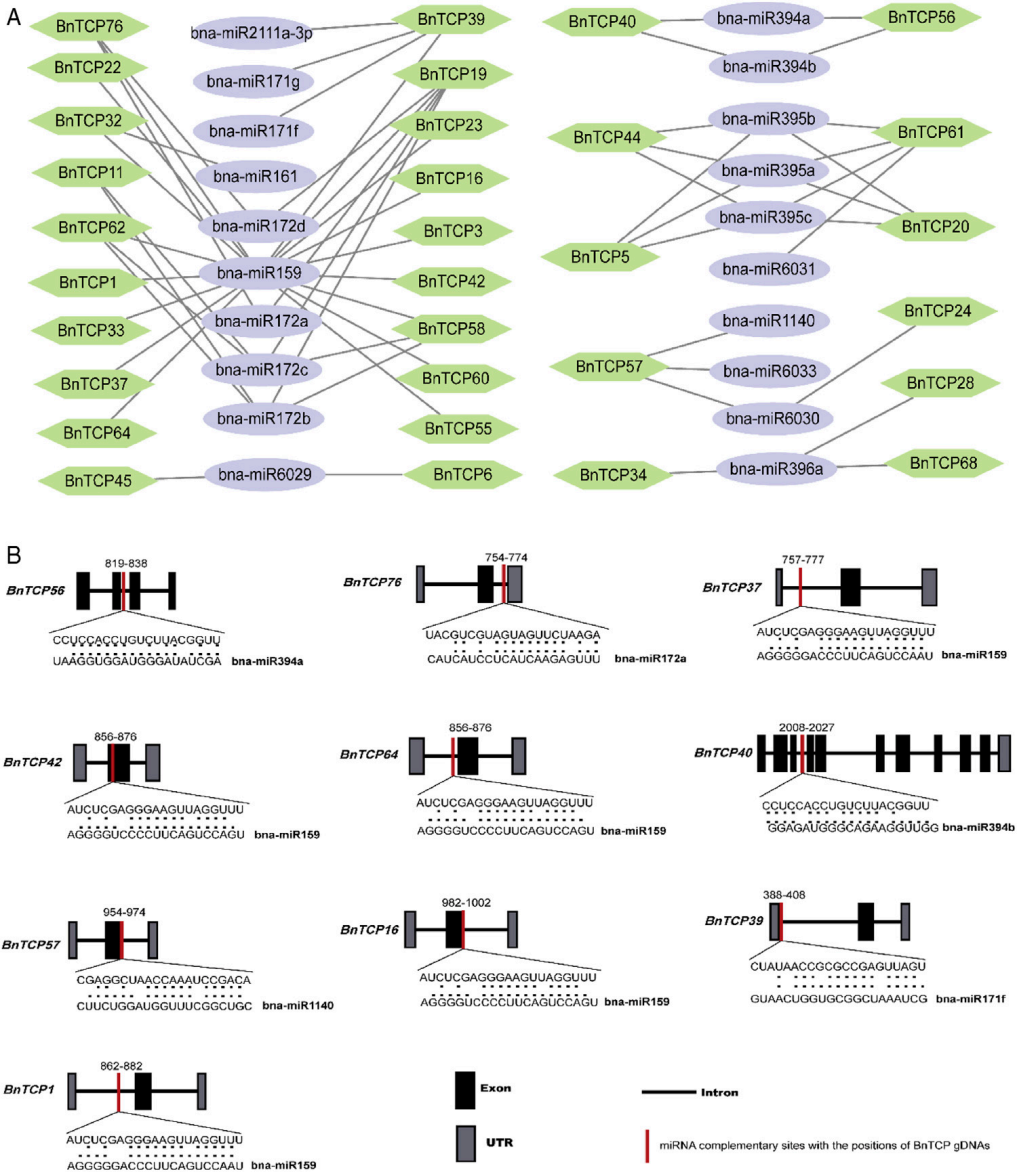
miRNA targeting *BnTCP* genes. **(A)** Network figure of anticipated miRNA targeting *BnTCPs* genes. Green hexagon colors correspond to *BnTCPs* genes, and bluish ellipse shapes represent miRNAs. **(B)** The schematic illustration signifies the *BnTCPs* targeted by miRNAs. The RNA sequence of each complementary site from 5′ to 3′ and the predicted miRNA sequence from 3′ to 5′ are exposed in the long-drawn-out areas. See Supplementary Table S7 for the detailed data of all predicted miRNAs.

During the past few years, various investigations have uncovered that miRNA-induced regulation participates in the stress responses in plants. Consequently, for a profound knowledge of miRNA-mediated post-transcriptional adjustment of *BnTCP genes*, we forecasted 32 miRNAs targeting 21 *BnTCP* genes (Figure 6A; Supplementary Table S7). Some of the miRNA-targeted sites are shown in Figure 6B, while the comprehensive data of all miRNAs targeted genes/sites is given in Supplementary Table S7. Overall, the results demonstrated that one member of the bna-miR1140 family targeted one gene (*BnTCP57*); one member of the bna-miR161 family targeted one gene (*BnTCP32*); one member of the bna-miR162 family targeted one gene (*BnTCP77*); one member of the bna-miR2111a-3p targeted one gene (*BnTCP39*); one member of the bna-miR6031 targeted one gene (*BnTCP61*); one member of the bna-miR6033 targeted one gene (*BnTCP57*); one member of bna-miR396 family targeted three genes (*BnTCP28, BnTCP34,* and *BnTCP68*); and one member of bna-miR159 family targeted 17 genes. Two members of the bna-miR171 family targeted one gene (*BnTCP39*); two members of the bna-miR6029 family targeted two genes (*BnTCP6* and *BnTCP45*); two members of the bna-miR6030 family targeted two genes (*BnTCP24* and *BnTCP57*); and two members of the bna-miR394 family targeted two genes (*BnTCP40* and *BnTCP56*). Three members of the bna-miR395 family targeted four genes (*BnTCP5, BnTCP61, BnTCP20*, and *BnTCP44*). Four members of the bna-miR172 family targeted five genes (*BnTCP11, BnTCP19, BnTCP58, BnTCP62*, and *BnTCP76*) (Figure 6A; Supplementary Table S7). In general, *BnTCP5, BnTCP19, BnTCP20, BnTCP39, BnTCP44, BnTCP57, BnTCP58, BnTCP61,* and *BnTCP76* were predicted to be targeted by a greater number (three and four) of miRNAs (Figure 6A; Supplementary Table S7). In further examination, the expression levels of these miRNAs and their targeted genes require validation to preside their biological functions in the rapeseed genome.

### Functional Annotation Study of *BnTCP* Genes

To further recognize the contribution of *BnTCP* genes, we performed GO annotation and enrichment analysis based on biological process (BP), molecular function (MF), and cellular component (CC) classes. These terms can boost our understanding of the diverse function of genes. The results of BP, MF, and CC exhibited several significantly enriched terms (Supplementary Table S8). For instance, in GO-BP class, 49 highly enriched terms were identified, including cellular process (GO: 0009987), RNA biosynthetic process (GO: 0032774), regulation of nucleic acid-templated transcription (GO: 1903506), heterocycle metabolic process (GO: 0046483), organic substance biosynthetic process (GO: 1901576), regulation of metabolic process (GO:0019222), nitrogen compound metabolic process (GO: 0006807) (Supplementary Table S8). The GO-CC enrichment outcomes distinguished ten highly enriched terms, including intracellular part (GO: 0044424), cell part (GO: 0044464), cellular_component (GO: 0005575), organelle (GO: 0043226), membrane-bounded organelle (GO: 0043227), intracellular (GO: 0005622), nucleus (GO: 0005634) (Supplementary Table S8). Similarly, the GO-MF results projected eight significantly enriched terms such as transcription regulator activity (GO: 0140110), molecular_function (GO: 0003674), DNA binding transcription factor activity (GO: 0003700), organic cyclic compound binding (GO: 0097159), nucleic acid binding (GO: 0003676) (Supplementary Table S8). Overall, GO enrichment analysis validates the functional role of *BnTCP* genes in several cellulars, molecular, and biological processes related to RNA/DNA binding, metabolic processes, transcriptional regulatory activities associated with rapeseed growth and developmental processes.

### Expression Profiles of *BnTCP* Genes Under Various Tissues

**FIGURE 7:**
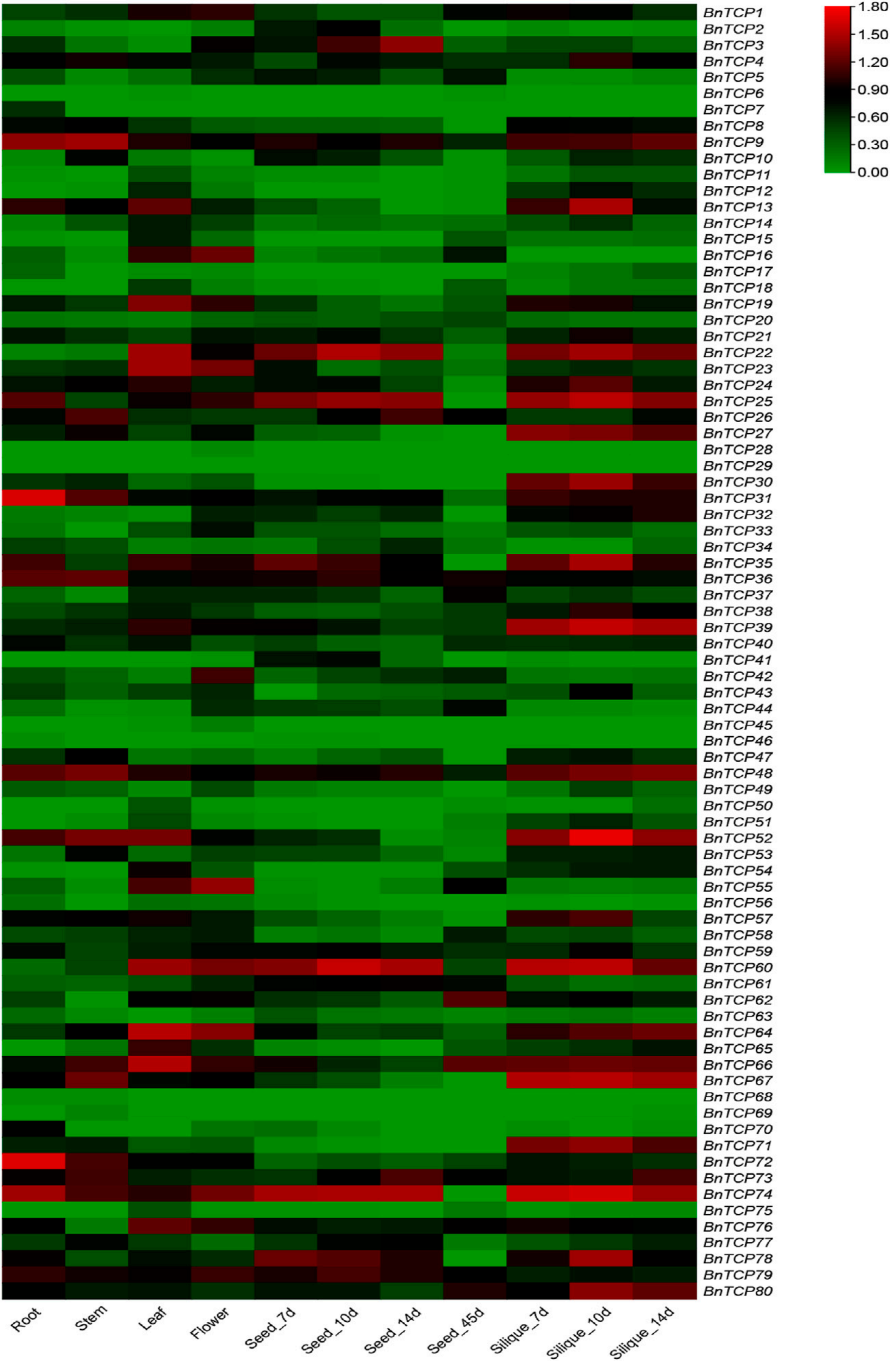
Expression profiling of *BnTCP* genes in various developmental organs/tissues. The 7, 10, 14, and 45 d tags specified the time-points when the samples were harvested. The red, black, and green colors display high to low expression levels. The expression heat map was created by taking log10 of transcripts per million (TPM) values.

Tissue-specific expression profiles of *BnTCPs* genes were examined in six diverse tissues and organs, i.e., roots, stems, leaves, flowers, seeds, and silique using RNA-seq data from *B. napus* (ZS11 variety) (BioProject ID: PRJCA001495). Overall, the results showed that only a few genes showed higher expression in some specific tissues (Figure 7). For instance, some genes showed higher expression levels in the root, including *BnTCP9, BnTCP13, BnTCP25, BnTCP31, BnTCP35, BnTCP36, BnTCP48, BnTCP52, BnTCP72,* and *BnTCP74*). In stem, a few genes showed higher expression levels, including *BnTCP9, BnTCP26, BnTCP31, BnTCP36, BnTCP48, BnTCP52, BnTCP66, BnTCP67, BnTCP72, BnTCP73,* and *BnTCP74*). In leaf, *BnTCP9, BnTCP13, BnTCP16, BnTCP19, BnTCP22, BnTCP23, BnTCP35, BnTCP39, BnTCP52, BnTCP55, BnTCP60, BnTCP64, BnTCP65, BnTCP66, BnTCP67, BnTCP74,* and *BnTCP76* genes display higher expression patterns. In flower, *BnTCP16, BnTCP19, BnTCP23, BnTCP35, BnTCP42, BnTCP55, BnTCP60, BnTCP64, BnTCP52, BnTCP55, BnTCP60, BnTCP64, BnTCP66, BnTCP74, BnTCP76,* and *BnTCP79* genes exhibited higher expression patterns. In seeds, several genes showed higher expression in seeds at different time points, such as *BnTCP3, BnTCP9, BnTCP22, BnTCP25, BnTCP35, BnTCP36 BnTCP60, BnTCP74, BnTCP78,* and *BnTCP79*). Likewise, in silique, many genes including *BnTCP9, BnTCP22, BnTCP24, BnTCP25, BnTCP27, BnTCP30, BnTCP31, BnTCP35, BnTCP39, BnTCP48, BnTCP52, BnTCP60, BnTCP64, BnTCP66, BnTCP67, BnTCP71, BnTCP74, BnTCP78,* and *BnTCP80,* showed higher expression patterns at different time points (Figure 7). Notably, some genes also showed moderate expression levels in various tissues. Overall, expression data show that only a few genes may significantly contribute to rapeseed growth and development. Therefore, the precise function of these genes could be discovered in future investigations.

### Expression Profiles of *BnTCP* Genes Under Phytohormones and Abiotic Stress Conditions

**FIGURE 8:**
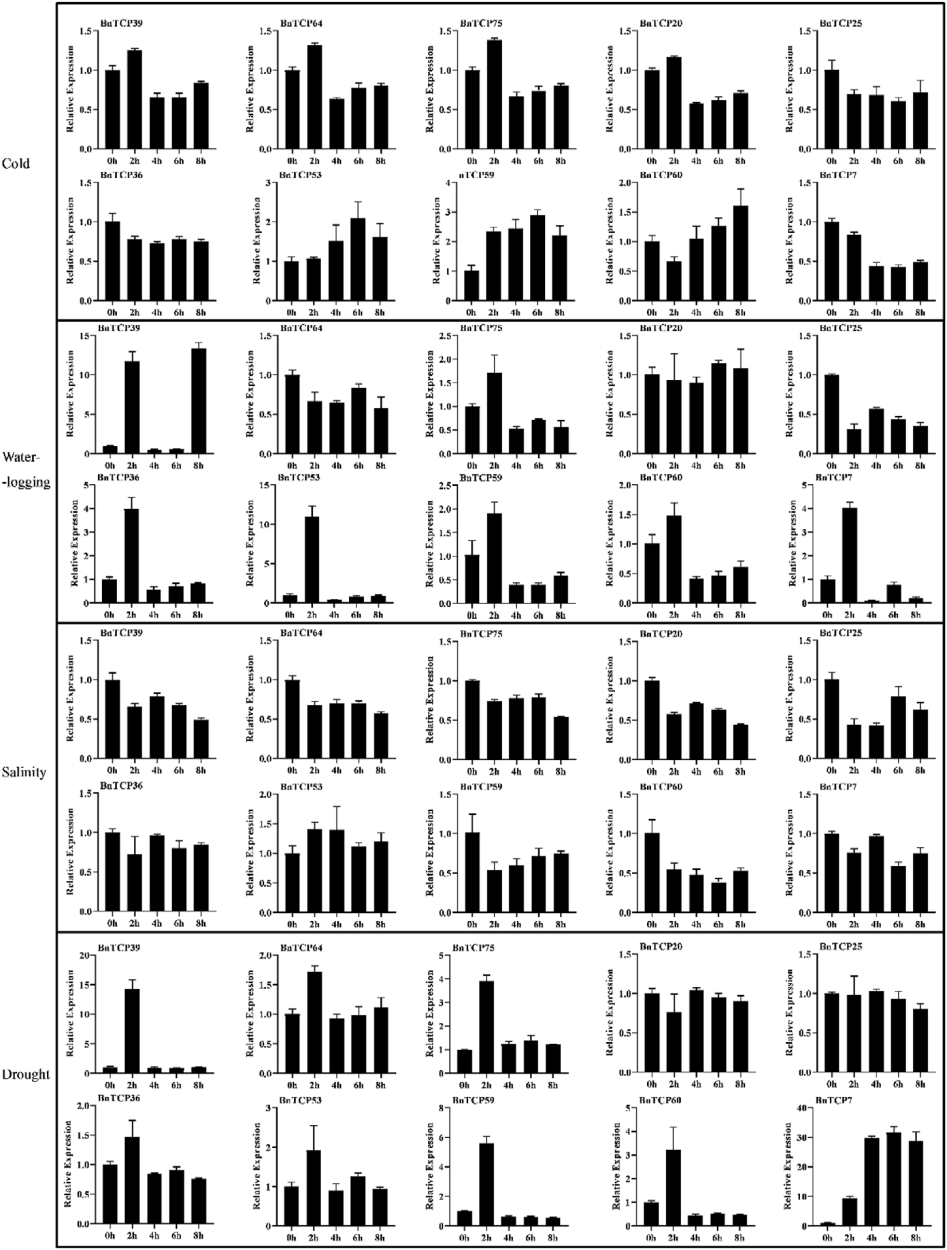
Expression patterns of the *BnTCP* genes under different abiotic stress conditions. The 0 h (CK), 2, 4, 6, and 8 h labels presented the time points (hours) when the samples were harvested for expression study after the stress treatment. Error bars represent the SD (*n* = 3).

**FIGURE 9:**
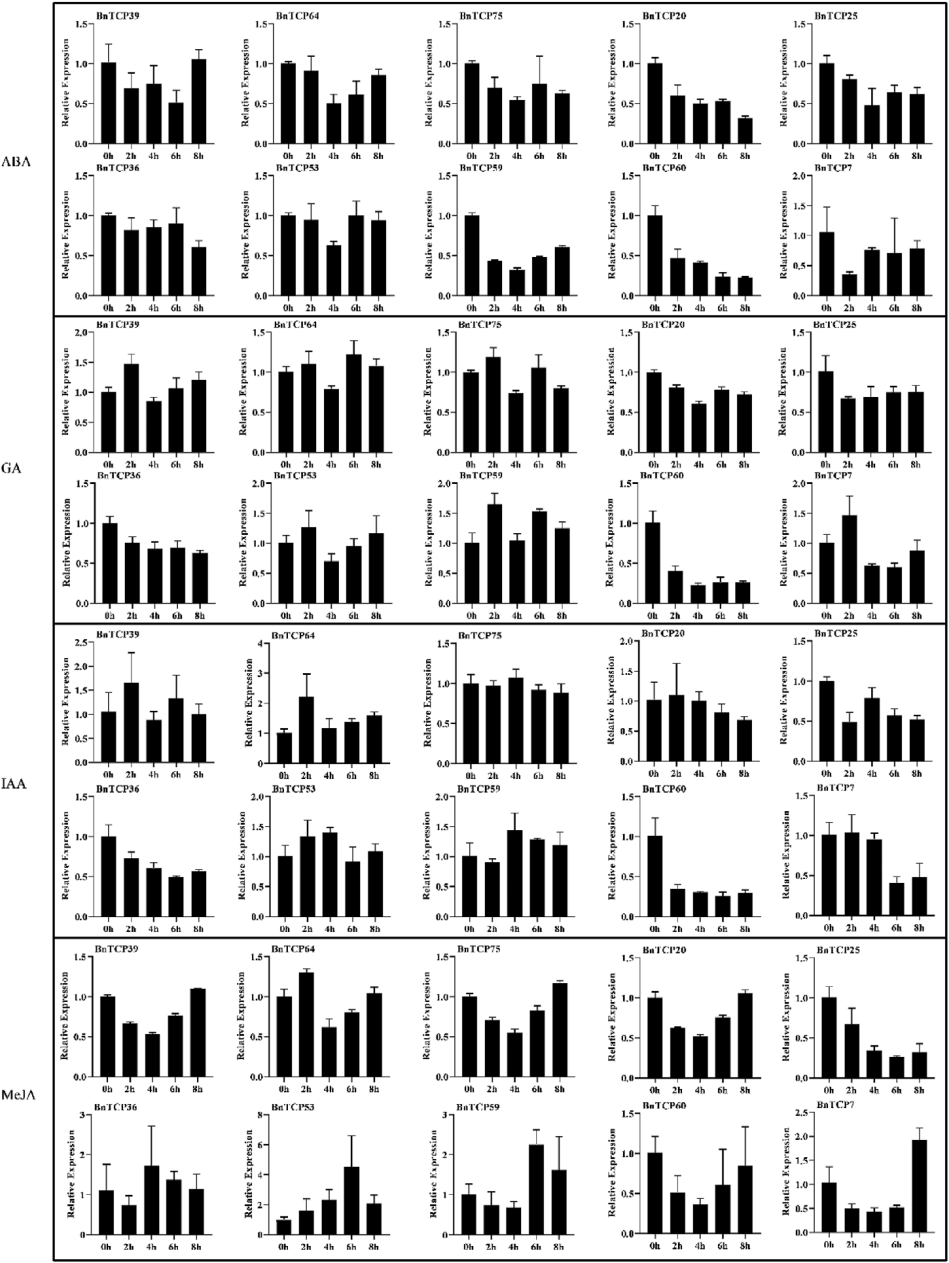
Expression patterns of the *BnTCP* genes under different hormones treatments. The 0 h (CK), 2, 4, 6, and 8 h labels presented the time points (hours) when the samples were harvested for expression study after the stress treatment. Error bars represent the SD (*n* = 3).

In this study, qRT-PCR-based expression profiles of ten randomly selected *BnTCPs* genes were evaluated in response to abiotic (cold, waterlogging, salinity, and drought), and phytohormones (ABA, GA, IAA, and MeJA) stresses at different time points (Figures 8, 9). Under abiotic stress conditions, most of the genes exhibited comparatively low expression levels, apart from some genes. For example, in response to cold stress, *BnTCP53* showed significantly higher expression at 4 h (1.521 fold), 6 h (2.081 fold), and 8 h (1.616 fold); *BnTCP59* showed higher expression at 2 h (2.312 fold), 4 h (2.409 fold), 6 h (2.873 fold), and 8 h (2.187 fold); and *BnTCP60* showed higher expression mainly at 6 h (1.254 fold), and 8 h (1.598 fold), compared to CK. Under waterlogging stress, *BnTCP39* showed higher expression at 2 h (11.684 fold), 8 h (13.369 fold); and *BnTCP53* showed higher expression at 2 h (10.865 fold), compared to CK. Under salt stress, almost all genes showed relatively higher expression at CK; however, *BnTCP53* display substantially higher expression at 2 h (1.402 fold), 4 h (1.395 fold), 6 h (1.109 fold), and 8 h (1.197 fold), compared to CK and other genes. In response to drought stress, only *BnTCP7* was up-regulated at 4 h (29.946 fold), 6 h (31.192 fold), and 8 h (28.428 fold) (Figure 8).

Under phytohormones treatment, almost all genes showed significantly higher expression except MeJA. Particularly, *BnTCP39, BnTCP64, BnTCP75, BnTCP25, BnTCP36, BnTCP53*, and *BnTCP7* display higher expression levels in response to ABA and GA treatments. These genes also possess ABA and GA-associated *cis*-elements in their promoter regions (Supplementary Table S6). Furthermore, *BnTCP39* under IAA treatment showed higher expression at 2 h (1.568 fold); *BnTCP64* significantly showed higher expression at 2 h (2.209 fold), 6 h (1.384 fold), and 8 h (1.590 fold) compared to CK. Notably, *BnTCP53* displays higher expression at 6 h (4.487 fold) in response to MeJA treatment than CK (Figure 9). However, *BnTCP60* did not reveal any significant expression to any stress conditions except cold stress, and *BnTCP53* was found to be more active in response to both hormones and abiotic conditions. These results are also consistent with the identified cis-elements in the promoter regions of genes.

## Discussion

### Characterization and Evolution of *TCP* Gene Family in Rapeseed

Rapeseed is an allotetraploid crop that practices broad genome replication and combination activities (Song et al., 2020). Nevertheless, rapeseed yield is pretentious by numerous environmental factors such as cold, heat, drought, salinity, and heavy metals toxicity (Raza et al., 2021a; Raza et al., 2021b; He et al., 2021; Raza, 2021; Su et al., 2021). The TCP TFs are antique plant-specific proteins (Cubas et al., 1999). So far, these TFs are yet to be reported in unicellular algae; and moss, pluricellular green algae, lycophyte, and ferns possess five-six *TCP* genes in their genomes (Martín-Trillo and Cubas, 2010). Owing to the development, duplications, and divergence of *TCP* genes, the gymnosperms and angiosperms plants possess bigger *TCP* gene families containing more than ten *TCP* members (Martín-Trillo and Cubas, 2010). Over the past few years, *TCP* gene families have been reported in various crop plants such as *Arabidopsis thaliana* (Yao et al., 2007), Moso bamboo (Liu et al., 2018), sweet potato (Ren et al., 2021), cucumber (Wen et al., 2020), rice (Yao et al., 2007), *Brassica rapa* (Du et al., 2017); *Brassica juncea* (He et al., 2020); and cotton (Ma et al., 2014; Yin et al., 2018), etc. To our best knowledge, the *TCP* gene family has not been fully characterized in the rapeseed genome. The accessibility of the rapeseed genome sequences offers resources for genome-wide identification of rapeseed genes (Song et al., 2020). Here, we recognized 80 *BnTCP* genes in the rapeseed genome. Notably, this is the largest *TCP* gene family found in the rapeseed genome due to whole-genome duplication (WGD; polyploidy). Previously, the highest number of *TCP* genes (73 members) was reported in allotetraploid cotton (Yin et al., 2018).

In the rapeseed genome, the *BnTCP* gene family may be evolved by WGD or segmental events, together with numerous tandem duplications. Notably, WGD differs from tandem duplication in that WGD enhances the quantity of all genes altogether and produces duplicate, theoretically redundant copies of all the genes within a genome. In contrast, tandem duplication is another vital way for gene expansion. Genes expanded by tandem duplication are always scattered all together as a cluster in chromosomes (Sémon and Wolfe, 2007; Fang et al., 2012). Gene duplication events of *TCP* genes were also spotted in several plant species (Ma et al., 2014; Lin et al., 2016; Yin et al., 2018; Wen et al., 2020). Apart from duplication events, exon-intron associations and numbers can also explain the evolutionary story of the gene family (Xu et al., 2012; Raza et al., 2021a; Su et al., 2021). The *BnTCPs* gene structure compared with the same group presented that *TCP* genes contribute to analogous exon/intron allocation in terms of exon and intron numbers; in the meantime, *BnTCPs* with the same class exhibited comparable motif patterns. These results imply that these *TCP* members may play identical roles in response to a variety of abiotic stresses. Similar observations have been reported in previous *TCP* gene families where both classes showed comparable diverse intron/exon distribution and motifs patterns (Ma et al., 2014; Lin et al., 2016; Yin et al., 2018; Wen et al., 2020).

### The Contribution of *TCP* Genes to Stress Responses and Tolerance Mechanisms

To get further insights into the contribution of *BnTCP* genes against various environmental cues, *cis*-elements in the promoter regions were envisaged. The findings demonstrated that two types of *cis*-elements were documented, i.e., stress (salt, drought, low-temperature, and anaerobic) and hormones (auxin, ABA, MeJA, and GA) associated. Consistent with earlier studies, *cis*-elements participate in the plant stress responses (Yamaguchi-Shinozaki and Shinozaki, 1994; Liu et al., 2014).

For instance, in rice, TCA-element between −563 and −249 bp upstream of *OsPIANK1* was found to be associated with resistance to *Magnaporthe oryzae* infection and exogenous SA treatment (Mou et al., 2013). ABREs are a class of *cis-*elements capable of binding to sturdily conserved ABA-dependent transcription factors, which exist in the promoter region of numerous stress-resistant genes and normalize the expression level of associated genes under abiotic stress conditions (Choi et al., 2000; Barbosa et al., 2013; Fujita et al., 2013). In wheat, the expression pattern of *TaGAPC1* was regulated under osmotic and salinity stress conditions. The promoter region of *TaGAPC1* contains various stress-related *cis*-regulatory elements (including MBS, DRE, GT1, and LTR), and methylation changes were observed during the stress treatments, leading to stress tolerance and regulation in gene expression (Fei et al., 2017). In the current study, these results were further authenticated by the GO annotation evaluation. For instance, GO enrichment analysis validates the functional role of *BnTCP* genes in several cellulars, molecular, and biological processes associated with RNA/DNA binding, metabolic processes, transcriptional regulatory activities related to rapeseed growth and developmental processes under stress conditions. Likewise, numerous scholars stated comparable outcomes in diverse crop plant species where *TCP* genes were found to play a substantial part in plant growth and developmental processes and responses to different abiotic stress episodes (Yao et al., 2007; Lin et al., 2016; Zhou et al., 2016; Wen et al., 2020). These results can enhance our knowledge of *BnTCP* genes under different stress conditions and rapeseed developmental processes at a molecular level.

Moreover, 10 randomly selected *BnTCP* genes’ expression profiling was appraised against various hormones and abiotic stress treatments. Particularly, *BnTCP7, BnTCP53,* and *BnTCP59* exhibited higher expression in response to cold, waterlogging, drought, and salinity stresses. Under hormones treatment, many genes showed higher expression, mainly by ABA, IAA, and GA treatments. These results are in agreement with recent reports where several *TCP* genes displayed higher expression levels under stress conditions. For example, in cotton, 41 *GhTCP* genes responded significantly to heat, salinity, and drought stresses (Yin et al., 2018). In cucumber, several *TCP* genes were up-regulated in response to photoperiod and temperature stresses; likewise, some genes were also induced by phytohormones like ethylene and gibberellin (Wen et al., 2020). In Moso bamboo, several *TCP* genes were substantially up-regulated in response to various hormones such as ABA, MeJA, and SA (Liu et al., 2018). The overexpression of maize *ZmTCP42* gene in *A. thaliana* led to sensitivity to ABA in seed germination and improved drought stress tolerance in transgenic *A. thaliana* (Ding et al., 2019). In another study, the overexpression of the *MeTCP4* gene regulated the cold tolerance by mediating ROS production and scavenging in transgenic *A. thaliana*. It also enhances the expression levels of stress- and ROS-scavenging-associated genes in the cold stressed *A. thaliana* plants (Cheng et al., 2019). Overexpressed *PeTCP10* gene improved salt and ABA tolerance of transgenic *A. thaliana* at the vegetative growth phase (Xu et al., 2021). These results indicate that *TCP* genes pointedly contribute to hormone signaling pathways and abiotic stress tolerance in various plant species.

### Expression Pattern of *TCP* Genes in Different Developmental Tissues

In the present study, tissue-specific expression profiles of 80 *BnTCPs* genes were assessed in six unique tissues using a publicly available RNA-seq dataset (https://ngdc.cncb.ac.cn/gsa/; BioProject ID: PRJCA001495). The aforementioned studies have illustrated that *TCP* genes may exhibit diverse expression profiles in various developmental tissues. For instance, the RNA-seq data was used to analyze the tissue-specific expression levels in cotton. The results showed that many *GhTCP* genes exhibited higher expression in various tissues, including mature leaves, stem, root, torus, petal, stamen, pistil, calycle (bracts), ovules, and fibers (Yin et al., 2018). In cucumber, qRT-PCR-based expression analysis showed that almost all 27 *CsTCP* genes exhibited higher expression in diverse tissues comprising root, stem, leaf, cotyledon, tendril, and flower (Wen et al., 2020). In *B. rapa*, some *BrTCP* genes showed higher expression in roots, leaves, and flower buds (Du et al., 2017). These results agree with our results where some *TCP* genes showed higher expression in all studied tissues (roots, stems, leaves, flowers, seeds, and silique), demonstrating that these genes may participate in rapeseed growth and development.

### miRNA: Key Performers in the Adaptation of Stress Responses

MicroRNAs (miRNAs) are a class of small-non-coding RNAs produced from single-strand hairpin RNA precursors. These miRNAs control gene expression through binding to complementary sequences within target mRNAs (Shen et al., 2015; Wani et al., 2020). Substantial development has been made in discovering the targets of rapeseed miRNAs that act to diverse stresses and also participate in developmental processes (Buhtz et al., 2008; Huang et al., 2010; Körbes et al., 2012; Zhou et al., 2012; Shen et al., 2015; Chen et al., 2018; Fu et al., 2019). In the present study, we anticipated 32 miRNAs targeting 21 *BnTCPs* genes. Recent reports support our results, e.g., five *TCP* genes, including *TCP2, TCP3, TCP4, TCP10,* and *TCP24,* were targeted by miR319 and have been involved in normalizing leaf morphogenesis in *A. thaliana* (Schommer et al., 2008). In cotton, three *TCP* (*GhTCP21, GhTCP31,* and *GhTCP54*) genes were targeted by miR319 (Wen et al., 2020). The overexpression of the miR319-associated *TCP* gene improves the growth, and it also increases the tolerance to salinity and drought stresses in transgenic bentgrass (*Agrostis stolonifera* L.) (Zhou et al., 2013).

The overexpression of soybean miR172c improves tolerance to drought and salinity stress, and it also enhances sensitivity to ABA in transgenic *A. thaliana* plants (Li et al., 2016). Recently, Cheng et al. (2021) found that miR172s (mainly miR172a and miR172b) act as a positive regulator of salt tolerance in rice and wheat. Further, it also maintains the ROS homeostasis during salt stress, generally by balancing the expression of several ROS-scavenging-related genes in both wheat and rice (Cheng et al., 2021). The transgenic *B. napus* plants overexpressing miR395 indicated a lower degree of cadmium-induced oxidative stress compared to wild-type plants (Zhang et al., 2013). Another miRNA (miR394) was reported to enhance the multiple abiotic stress tolerance, including salinity and drought (Song et al., 2013) and cold stress (Song et al., 2016), in *A. thaliana* plants by ABA-dependent manner. These discoveries stipulated that miRNA might play critical roles in plant growth, development, and abiotic stress tolerance by regulating the expression levels of their target and stress-responsive genes.

## Conclusion

In summary, for the first time, we identified 80 putative *BnTCP* genes in the rapeseed genome, which are distributed on 18 chromosomes. Genome-wide *in silico* analysis such as characterization, evolution, gene structures, conserved motifs, *cis*-elements, miRNA prediction, and GO annotation was performed to gain insights into *TCP* genes in rapeseed. Furthermore, their expression profiling was also appraised in different tissues and against diverse hormones and abiotic stresses. In a nutshell, this study laid the basis for additional functional studies (such as overexpression, knockout *via* CRISPR/Cas system, etc.) of the *BnTCP* genes in rapeseed breeding programs under adverse environmental conditions.

## Data Availability

The original contributions presented in the study are included in the article/Supplementary Material, further inquiries can be directed to the corresponding authors.
