# Supplementary material for: Comprehensive In Silico Characterization and Expression Profiling of TCP Gene Family in Rapeseed
Source: Front Genet. 2021 Nov 17;12:794297. doi: 10.3389/fgene.2021.794297 (PMC8635964; doi:10.3389/fgene.2021.794297)
Supplement: Supplementary file 1 [file DataSheet1.ZIP › Supplementary Table S1.docx]

**Table S1.** A list of primers used for gene expression analysis by qRT-PCR.

| **Primer name** | **Primer sequence** |
| --- | --- |
| BnActin_F | CTGGAATTGCTGACCGTATGAG |
| BnActin_R | ATCTGTTGGAAAGTGCTGAGGG |
| BnTCP39_F | TTTCGTCTTCTTATTGCAGGGC |
| BnTCP39_R | CTTCGTTTAAGCTTGTCTGCAA |
| BnTCP64_F | CTTATGCACAGCAACTACCATC |
| BnTCP64_R | CAAGCCTCTGAATTGACTGTTG |
| BnTCP75_F | ACAGCAATTCAACTTTACGACC |
| BnTCP75_R | GTTAAAGCCATGTGGGAATTGT |
| BnTCP20_F | TCAAGGTCTCAACTTGCAAATG |
| BnTCP20_R | AAGAGCCATTGAAATCAAACCC |
| BnTCP25_F | ACTATTTCCACTTCCACGAACT |
| BnTCP25_R | GAATCCTCTCTCAGGCGTTTTC |
| BnTCP36_F | TATAGGTTTGAGTATGCCGGAC |
| BnTCP36_R | TTACATCAATCACCCATCGCTA |
| BnTCP53_F | TTCTTGGTTCAAAACGTAGCTG |
| BnTCP53_R | CTCTGAGCATATTCTTCGTTGC |
| BnTCP59_F | TGTATCAGTTCCTGACGTTACC |
| BnTCP59_R | AGCTCCTGTTTCTCGTAAATCT |
| BnTCP60_F | GGAGAAGAACCTACTCAACCAA |
| BnTCP60_R | TTCCTCCGTGTCATGATAGATG |
| BnTCP7_F | CTAGAGAACGAGCAAGAGAGAG |
| BnTCP7_R | AGGTTTTCTCTTTCTTGTGCAC |
